# Advancing malware imagery classification with explainable deep learning: A state-of-the-art approach using SHAP, LIME and Grad-CAM

**DOI:** 10.1371/journal.pone.0318542

**Published:** 2025-05-28

**Authors:** Sadia Nazim, Muhammad Mansoor Alam, Syed Safdar Rizvi, Jawahir Che Mustapha, Syed Shujaa Hussain, Mazliham Mohd Suud

**Affiliations:** 1 Malaysian Institute of Information Technology, Universiti Kuala Lumpur, Kuala Lumpur, Malaysia; 2 Department of Computer Science, Bahria University Islamabad, Islamabad, Pakistan; 3 Faculty of Computing, Riphah International University, Islamabad, Pakistan; 4 Department of Computer Science, Bahria University Karachi, Karachi, Pakistan; 5 Department of Computer Science, Sir Syed CASE Institute of Technology, Islamabad, Pakistan; 6 Multimedia University, Persiaran Multimedia, Cyberjaya, Selangor, Malaysia; Mehran University of Engineering & Technology, PAKISTAN

## Abstract

Artificial Intelligence (AI) is being integrated into increasingly more domains of everyday activities. Whereas AI has countless benefits, its convoluted and sometimes vague internal operations can establish difficulties. Nowadays, AI is significantly employed for evaluations in cybersecurity that find it challenging to justify their proceedings; this absence of accountability is alarming. Additionally, over the last ten years, the fractional elevation in malware variants has directed scholars to utilize Machine Learning (ML) and Deep Learning (DL) approaches for detection. Although these methods yield exceptional accuracy, they are also difficult to understand. Thus, the advancement of interpretable and powerful AI models is indispensable to their reliability and trustworthiness. The trust of users in the models used for cybersecurity would be undermined by the ambiguous and indefinable nature of existing AI-based methods, specifically in light of the more complicated and diverse nature of cyberattacks in modern times.

The present research addresses the comparative analysis of an ensemble deep neural network (DNNW) with different ensemble techniques like RUSBoost, Random Forest, Subspace, AdaBoost, and BagTree for the best prediction against imagery malware data. It determines the best-performing model, an ensemble DNNW, for which explainability is provided. There has been relatively little study on explainability, especially when dealing with malware imagery data, irrespective of the fact that DL/ML algorithms have revolutionized malware detection. Explainability techniques such as SHAP, LIME, and Grad-CAM approaches are employed to present a complete comprehension of feature significance and local or global predictive behavior of the model over various malware categories. A comprehensive investigation of significant characteristics and their impact on the decision-making process of the model and multiple query point visualizations are some of the contributions. This strategy promotes advanced transparency and trustworthy cybersecurity applications by improving the comprehension of malware detection techniques and integrating explainable AI observations with domain-specific knowledge.

## Introduction

The amalgamation of AI in our day-to-day life is getting more prevalent. As stated by a quantitative evaluation performed by Grand View Research, the global AI market was estimated at USD 93.5 billion in 2021, with an estimated compound annual growth rate (CAGR) of 38.1% from 2022 to 2030. AI is now being exploited considerably not only in the field of cybersecurity but also in several other realms. The global cybersecurity industry was valued by Mordor Intelligence to cost $156.24 billion in 2020 and is expected to multiply at a yearly rate of 14.5% to $352.25 billion by 2026 [1].

It is significant that, in 2021, worldwide cyberattacks increased by 29%, as stated by the 2021 Cyber Trends Report, even though there were approximately 0.3 billion more internet users globally in 2021 than there were the preceding year. An attack on an IT company in June 2022 restricted unemployment compensation and job-search assistance to hundreds of people in several states in the United States, which will lead to severe social instability during the COVID-19 epidemic. As stated in a paper published by the European Union Agency for Network and Information Security (ENISA) [2], It is estimated that the safety and reliability of digital technology will become even more important under the newly established ethical and social standards created by the COVID-19 pandemic [3]. These statistics and results reveal the significant points that cyberspace and associated systems and machines have experienced more hackers and intruders these days [1].

Thus, to ensure the confidentiality, accessibility, and authenticity of data exchanged over the World Wide Web, a trustworthy and safe cybersecurity technology needs to be developed. The constantly increasing content distributed via cyberspace poses challenges for conventional signature-based and rule-based cybersecurity solutions. However, cyber attackers persistently exploit distinctive, manipulative, and sophisticated attack tactics and utilize progressive technology, such as artificial intelligence, to keep themselves a step ahead of law enforcement and augment their malicious behavior’s complexity and efficacy [4]. Accordingly, to boost the efficacy of cyber protective frameworks, cybersecurity investigators are now concentrating on artificial intelligence-based techniques, specifically ML and DL, in addition to more typical (non-AI) safety measures like game theory, rate control, and autonomous systems [3].

The implementation of AI techniques, particularly ML and DL algorithms, has presented potential in a variety of cybersecurity domain programs, such as intrusion detection, spam e-mail filtering, botnet detection, fraud detection, and malicious application identification. Nonetheless, these methods have no deficiencies, which are more costly than conventional cybersecurity measures [5]. Conversely, some cybersecurity technologists have composed their frameworks to be more intricate and difficult to comprehend to attain better precision at the cost of interpretability. The European Union’s General Data Protection Regulation has exposed this lack of comprehensibility, preserving the capacity to comprehend the rationale underlining an AI algorithmic decision that has an adversarial effect on people [6].

AI algorithms experience vagueness, which makes it difficult to comprehend their underlying operational mechanisms, even when they seem effective in outcomes and predictions. In an area like cybersecurity, this attribute illustrates the more challenging situations since it presents obvious hazards to designating significant decision-making responsibility to a technique vulnerable to self-justification [7]. Considering this state, XAI endorses progressing toward more interpretable AI to overcome this issue. XAI methods intend to develop approaches that contribute to outstanding accuracy and prepare more interpretable frameworks. Hence, to trust the evaluations made by cybersecurity frameworks, AI must be understandable and explainable. To fulfill these requirements, several approaches have been offered to make AI decisions more comprehensible to humans and these explainable techniques are usually shortened as “XAI” [1].

### Challenges, motivation and contribution

A broad range of systems powered by AI can be utilized to identify malware with a spectrum of explainability; yet, many of these approaches have been designed for non-imagery data and consequently do not effectively tackle specific challenges inherent in malware imagery analysis. Malware imaging data involves sophisticated approaches with the capacity to address both reliable classification and comprehensibility, considering its diversified behavioral characteristics across multiple virus families. The currently available explainability approaches provide certain information but have constraints in their utilization; they generally only offer limited explanations that might not fully capture the diverse nature of malware imaging. Furthermore, a diverse approach is required to facilitate more complex and multidimensional explainability since distinct types of malware vary dramatically in appearance and behavior. This involves developing solutions that are competent to present layered interpretations toward various malware attributes in addition to performing an extensive evaluation of malware images, confirming that local and global interpretations are offered. Bridging this gap is critical for constructing accurate frameworks that experts may use in high-stakes cybersecurity scenarios.

The motivation behind this research is to accommodate the rising demand for robust and transparent AI-based solutions in malware imagery analysis. Since cyberattacks are becoming increasingly prevalent and advanced, it becomes essential that researchers understand the rationale behind the underlying malware classification decisions to formulate substantial protections. The ultimate goal of the present investigation is to address the explainability of the reliability gap by employing interpretable approaches that might boost model transparency without compromising accuracy. The present study aims to provide comprehensive insights using SHAP, LIME, and Grad-CAM so cybersecurity practitioners can fully understand model behavior, develop reliance on automated solutions, and make wise decisions in digital security scenarios.

The current research proposes an innovative approach to malware classification that beats out previous approaches by integrating an ensemble deep neural network with a blended malware dataset. This approach incorporates the MalImg and MaleVis datasets to develop a wide-ranging, diversified dataset that optimizes model perseverance, in contrast to prior investigations that typically involve distinct datasets or basic neural networks. The technique is particularly advantageous for managing the complications of malware detection with higher efficiency and comprehensibility since the ensemble deep neural network framework leverages multiple algorithms to enhance classification accuracy. It leverages SHAP, LIME, and Grad-CAM for comprehensibility, introducing an explainability with the best predictive framework. This research strengthens global and local system observations by incorporating these comprehensibility approaches, delivering a deeper understanding of the decision-making processes involved in malware identification and classification. This methodology fills the highlighted research gap by demonstrating the distinctive insights that each explainability methodology contributes in addition to establishing that integrating numerous explainability techniques is feasible. The research improves this area toward more secure and reliable digital security by paving a road map for the development of DL approaches in malware analysis that are more transparent, interpretable, and trustworthy.

### Novelty of study

The following key aspects are utilized to highlight the novelty of this study:

1. The key domain is to comprehensively deal with the explainability gap in malware classification techniques to develop specifically on malware imagery data.
2. The best-performing framework for explainability is identified by comparing ensemble DNNW with different ensemble strategies, including RUSBoost, Random Forest, Subspace, AdaBoost, and BagTree.
3. The XAI application implements SHAP, LIME and Grad-CAM to give a deep neural network model a variety of explainability viewpoints.
4. Interpretability is boosted by the extensive, visually accompanied study of feature importance spanning several query points delivered by detailed feature importance analysis.
5. Promotes the alignment of XAI findings with domain knowledge, producing more transparent and trusted malware detection solutions.

## Background

**Fig 1:**
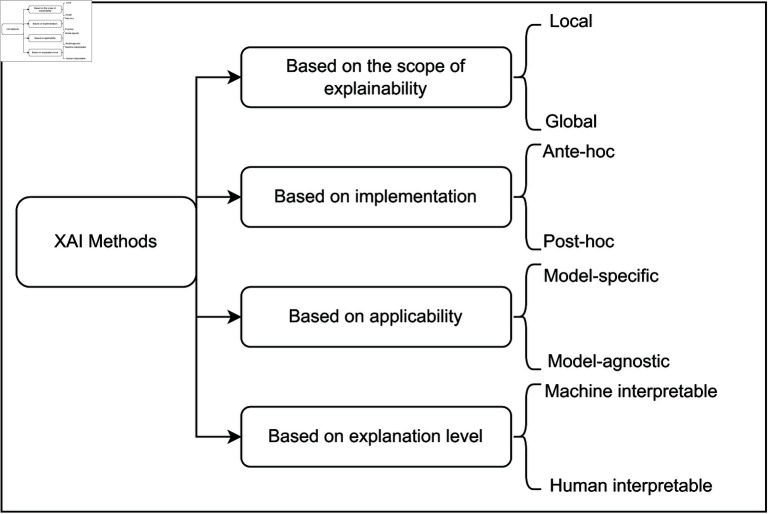
Taxonomy categorizes the various XAI approaches and methods.

A systematic categorization of the different strategies and methodologies that can be employed to explain an AI model has been developed gradually. The key point to note is the difference between explainability and interpretability. Interpretability is about determining the relationship between causes and impacts inside an AI system. However, explainability extends beyond interpretability by delivering a human-understandable explanation of the methodology and justification behind a prediction-making process of the model [7]. A meaningful transformation of real comprehensibility (interpretable models) and posthoc explanations (supplementary techniques to shed interpretability on state-of-the-art black-box frameworks) is prepared in Fig 1, which also demonstrates the existing taxonomy. These techniques entail producing local explanations for precise inputs or the global understanding of the performance of the model [1]. Here’s a brief synopsis:

**Interpretability/explainability:** If the performance of the model is considered sufficient during the evaluation phase, the subsequent phase is to comprehend or justify the classification findings to the level that the classification is understandable and explicable. Different models have been exploited to imitate real-world data; however, the explanation of possibly critical data depends on the input. The forecasting engine examines the findings using features, graphs, and images. There are two potential methods to produce these justifications: either local rationales generated during the testing stage or global rationales that adjust the parameters of the forecasting engine during the training phase [1, 8]. The following section proposes more knowledge on these various categories of explanations.

### XAI categorization

The XAI frameworks are classified into four classes based on interpretability.

1. **Model-Specific or Model-Agnostic:**
  **Model Specific:** It regulates whether the inference approach is restrained to a specific framework or not. The strategies and techniques that are exclusive to such a framework are known as model-specific. **Model Agnostic:** Any ML methods can be formulated interpretably by using model-agnostic techniques. These frameworks cannot view the internal model data, such as weights or structural parameters [4].
2. **Intrinsic and Extrinsic (posthoc):** These terms encompass whether a model may be explained autonomously or if approaches that investigate frameworks after training are indispensable to achieve interpretability. Straightforward and transparent frameworks such as decision trees are intrinsic. Interpretability is attained indirectly by applying an interpretation technique after training is extrinsic [1].
3. **Local or Global Interpretation:** The capacity of the explanation method, whether it relates to a single data record or the behavior of the whole model, is categorized as local or global [9]. Global techniques and tools explain the entire model, whereas local approaches only interpret a single prediction [6].
  **Global interpretation:** It inspects the framework systematically, highlighting its universal properties, frameworks, and illustrations. This type of understanding provides insight into the dynamics of the complete model beyond every statistic. It evaluates the degree of weights and biases of models that contributed towards predictions. In a nutshell, it exhibits the allocation of the estimated results regarding the attributes. For example, global interpretation may incorporate evaluating the influence of different layers and neurons on the forecasting process in a neural network trained to classify malware. For global interpretation, techniques like feature significance scores are commonly utilized, which measure the involvement of each feature in the final forecast [6]. However, as the magnitude of hyperparameters increases, it becomes more challenging to achieve global interpretability, specifically when working with feature domains that are more substantial than three dimensions. For example, consider a DL model employed to classify malware by utilizing a variety of static and dynamic variables as training data [10, 11]. The total significance of features like PE headers, API calls, and network traffic patterns are investigated using a global interpretation technique. The framework might be made to demonstrate that API requests have a greater influence on predictions than PE headers by using feature importance scores. The framework is developed based on significant scores of features, demonstrating that API requests have a larger impact on predictions than PE headers [4, 8].
  **Local interpretation:** The key objective of local interpretation is understanding the prediction of the model for a single instance. This method helps to describe the justification behind the prediction made by the model. For example, in the case of malware classification and detection, local interpretation could notice the specific features of a malware sample (like particular API calls or byte sequences). The model mostly utilizes these features to determine malicious samples. Shapley Additive Explanations (SHAP) and Local Interpretable Model-agnostic Explanations (LIME) are preferably used to perform the local interpretation. These methods fabricate explanations and expose the most influential features in the prediction [8]. For example, SHAP values compute the impact of each feature determined by cooperative game theory, while LIME may disturb the input features and watch changes in the outcome of the model to find key features. Consider a malware sample that is classified as malicious by the framework. The local explanation performed by SHAP analysis highlights those features that have a great impact on the decision-making process of the model. For example, the justification can explain the reason behind the significance of odd API calls and particular byte sequences in the malware classification that are crucial in recognizing it as malicious software [12].

### XAI frameworks and methods

Explainable AI approaches can be categorized into different groups based on the types of justifications (feature attribution, rules, or counterfactual) they delivered:

1. **Local Interpretable Model-agnostic Explanations (LIME):** The explanation of the decisions made by a DL model can be performed by employing LIME, a model-agnostic explanation technique. It performs operations in two steps: Initially, it sympathetically disturbs an input instance to generate many illustrations. The subsequent step implicates directing the illustrations to the black-box model, which then employs the prediction to train an intermediary framework—typically a decision tree or linear regression that is inherently understandable. The final justifications of the input framework are originated from the parameters of the interpretable model [12].
2. **Shapley Additive Explanation (SHAP):** utilizes a game theory methodology to render justifications. It utilizes Shapley values for feature identification; however, it is an approximation method like LIME. The Shapley value characterizes the average insignificant impact of a feature across all potential arrangements or feature combinations used to make a prediction. Shapley values satisfy the specifications of local accuracy and stability, which are fundamental traits for feature identification [1].
3. **Gradient:** The association between input attributes and output is rationalized by the gradient approach. It leverages the information movement in a neural network during backpropagation. It computes the gradient of the prediction performed by the model concerning the input [6, 13].
4. **Integrated Gradient:** The integrated gradient technique involves identifying the gradient of the output class against the input. However, important features are offered minimal magnitudes in some situations while classifying class scores. This causes an impact on the training process saturation of deep neural networks. The integrated gradient technique employs gradients individually to deal with this situation. It aggregates gradients along a linear path from the initial point to a specific test instance. Based on the use case, an initial point is determined. By aggregating the values, this method estimates the percentage that each input contributes to the outcome [14, 15].
5. **DL Important Features (DeepLIFT):** This method calculates feature identification scores by evaluating the stimulation of each neuron to a benchmark activity. Similar to integrated gradients, this technique determines appropriate stimulations based on the particular use case. Identifying a suitable stimulation is essential for the accurate computation of feature attribution [1].
6. **Occlusion:** This technique is a deviation-based local explanation approach that successively substitutes input features with a fixed value, commonly zero, to calculate feature significance. The relevance of a feature to the training model is established by the degree of accuracy reduction examined when the feature is substituted. The justification explained earlier delivers the most significant features discovered by the model as output when applied to a machine-learning framework. Each explanation method yields a feature explanation based on its fundamental principle of operation. These feature assessments are indispensable in understanding the learning capacity and reliability of the framework [1].

## Research problem

The evolution of malware offers a serious hazard to cybersecurity since new variations are continuously evolving. To address this risk significantly, malware must be precisely classified into families [16]. This approach accelerates a thorough understanding of the behavioral dynamics of threats and facilitates the advancement of powerful defensive techniques. Both victims and security professionals are obscured concerning the underlying designs and patterns that explain the reason behind the sample malware association to a particular family. That is why traditional malware detection techniques often prove unsatisfactory in this context. Hence, the key challenges and issues, specifically highlighting the limited explainability in existing AI-based malware detection methods, especially for imagery data.

## Literature review and research gap analysis

Much advancement has been achieved in malware identification, particularly due to the advent of the ML and DL approaches. However, a shortage of investigations persists on how interpretability could be integrated into these models [17, 18]. A significant amount of present work focuses on standard, non-imagery malware data to achieve excellent identification and categorization accuracy by leveraging features retrieved through binary or textual analysis. These endeavors have led to powerful classifiers, but much less attention has been focused on how interpretable these frameworks are, specifically when addressing malware imagery. The comprehensibility of malware imagery data is significantly deficient, as Table 1 demonstrates since the majority of comprehensible research currently available is based on non-imagery data. Although ML/DL methodologies have been employed in studies like [19–21] for image-based malware detection and classification, they are not fully understandable. In comparison with [20, 21], which performs image-based malware classification without any explainability, [19] uses Grad-CAM, which provides minimal comprehensibility. However, there is preliminary work on malware imagery investigation, but it lacks adequate explainability, revealing a major research gap. This is highlighted by the integration of research articles [20, 21] in the present study. Bridging this gap is critical to establishing more transparent and trusted malware detection systems in progressively digital environments. This present investigation bridges this gap by presenting the first comprehensive investigation of malware image datasets that combines interpretability techniques like LIME, SHAP, and Grad-CAM with a high rate of classification. This article renders a novel contribution to this discipline by addressing both the explainability and performance of these models laying the ground for more explainable and transparent malware classification methods [22, 23].

**Table 1:** Summary of existing literature on explainability in malware classification.

Reference	XAI Technique	ML/DL Frameworks	Datasets	Accuracies
[24]	LIME	Support vector machine (SVM), Multi-layer perceptron (MLP), KNN, RF	AndroZoo, Drebin, XMal and Fan et al	99.27%
[25]	LIME, SHAP	–	–	94%
[26]	Lime, SHAP	CNN	VirusShare, RD1 (Ransomware Dataset 2)	99.4%
[27]	SHAP	(CNN) and Bi-Gated Recurrent Unit (Bi-GRU) Android Malware Detection (AMD)	CICAndMal2019	97.76%
[28]	LIME	RF, XGBoost, Logistic Regression, LSTM, GRU, ANN, and hybrid models	CIC-MalMem-2022	87.30%
[29]	SHAP	Decision Tree/Entropy (DT/Entropy), Decision Tree/Gini (DT/Gini), KNN, Lightweight Gradient Boosting Machine (LGBM), LR, and SVM	Evasive-PDFMal2022	99.9%
[30]	LIME, SHAP	DNN, Alexnet and LeNet	HPC dataset, Mimicus dataset	93%
[19]	Grad-CAM	GNB, SVM, DT, LR, K-NN, and ensemble classifiers with CNN models	R2-D2 IoT Device Dataset, MalNet IoT Device Dataset	96%
[31]	LIME, SHAP	DT, SVM, CNN, , GNB, SVM, and LR	CIC-InvesAndMal2019	good
[20]	–	DL, ML	Malimg, Microsoft BIG 2015, and Malevis	97.78%
[21]	–	CNN-based	Malimg	99.44%

## Dataset and experimental analysis

This research leverages a blended malware dataset to provide robust malware classification as well as the interpretability and explainability of the predictive framework. The study presents a comprehensive collection of malware images as visual representations of binary files by integrating the MalImg and MaleVis datasets, which renders it feasible to visually evaluate and categorize them using deep learning techniques [31]. The amalgamated dataset serves as a strong basis for training ensemble deep neural networks and other ensemble methods for obtaining the best classification accuracy since it encompasses both balanced and imbalanced class populations. However, the understanding and comprehensibility of the algorithm’s decision-making process are among the primary areas to investigate for this research. The most promising predictions from the deep ensemble framework are thus comprehended using three explainable AI techniques: Grad-CAM, LIME, and SHAP.

### Performance evaluation

The blended malware dataset has been categorized by employing the ensemble models, which include ensemble deep neural network [33], RUSBoost, Random Forest, Subspace, AdaBoostM2 and Bagging as baseline classifiers. These frameworks have been chosen since they may enhance predicted accuracy by joining several weak learners to generate a more robust framework. This is very beneficial when addressing the intricate and unpredictable nature of malware data. Such ensemble techniques perform effectively with a dataset like this because they can substantially and effectively improve accuracy by identifying patterns in the data and managing imbalances. Establishing these robust baselines before using explainability approaches such as SHAP, LIME and Grad-CAM is fundamental to guarantee that the interpreted models are reliable and effective in their predictions.

The Table 2 estimates the performance evaluation of several ensemble models to determine how well they perform when pertained to the same dataset. The Ensemble Deep Neural Network [32] obtains the largest accuracy of 96.06%, demonstrating the effectiveness of DL models in classifying intricate patterns in malware data. RUSBoost and BagTree also demonstrate robust performance with accuracy rates of 94.23% and 93.45%, respectively. Progressing generalization and addressing class imbalance can be achieved with these strategies. However, the Subspace technique demonstrates a substantially lower accuracy of 73.50%, representing that it may not be as suitable for this specific dataset. The average performance of the AdaBoostM2, with an accuracy of 87.42%, denotes that despite being adaptable, it may not be able to detect every detail of the data as efficiently as other techniques. The accuracy of the Random Forest is 93.35%, which matches meticulously with the performance of RUSBoost, highlighting its strength as an ensemble method. The prevailing work referenced displays a comparable efficiency level with precision of 95%, which recommends that the proposed ensemble deep neural network, offer an advancement over conventional approaches.

**Table 2:** Performance evaluation of ensemble models through hyper-parameteric values and accuracies.

Ensemble Models	Hyper-parameters	Accuracies
Ensemble Deep Neural Network	Epochs: 500, Batch Size: 150, Learning Rate: 0.003	96.06%
RUSBoost	Number of Learning Cycles: 4000, Learning Rate: 0.004	93.45%
Random Forest	numTrees:3000	93.35%
Subspace	Number of Learning Cycles: 3000	73.50%
AdaBoostM2	Number of Learning Cycles: 3000, Learning Rate: 0.1	87.42%
BagTree	Number of Learning Cycles: 3000	94.23%
Existing Result [31]	–	95%

**Fig 2:**
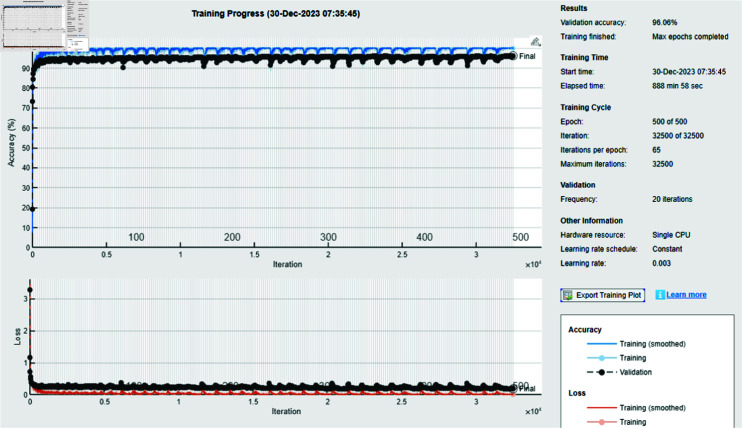
Training results of deep neural network for imagery dataset by considering 0.003 learning rate and 500 epochs.

**Fig 3:**
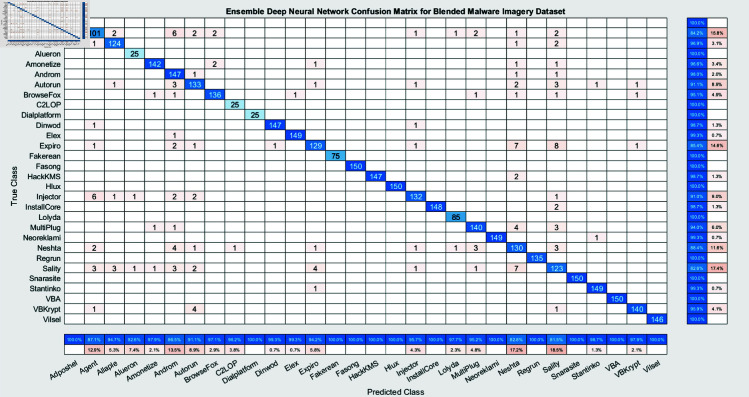
Confusion matrix for ensemble deep neural network illustrates the true positives, false positives, true negatives, and false negatives for each class.

**Fig 4:**
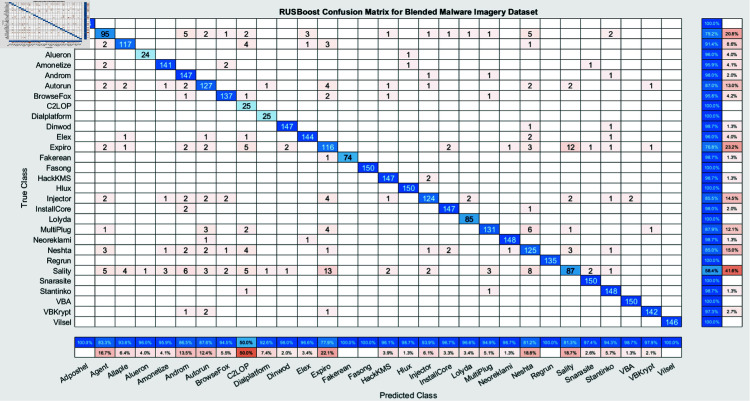
Confusion matrix for RUSBoost algorithm (3000 Learning Cycles) highlights the classification accuracy across different classes.

**Fig 5:**
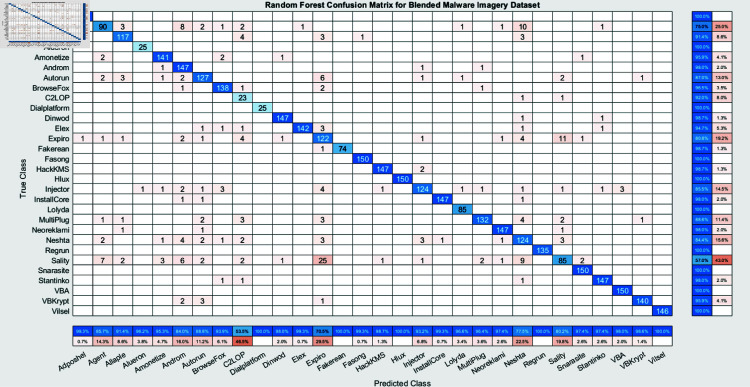
Confusion matrix for Random Forest algorithm (3000 Number of trees) highlights the classification accuracy across different classes.

**Fig 6:**
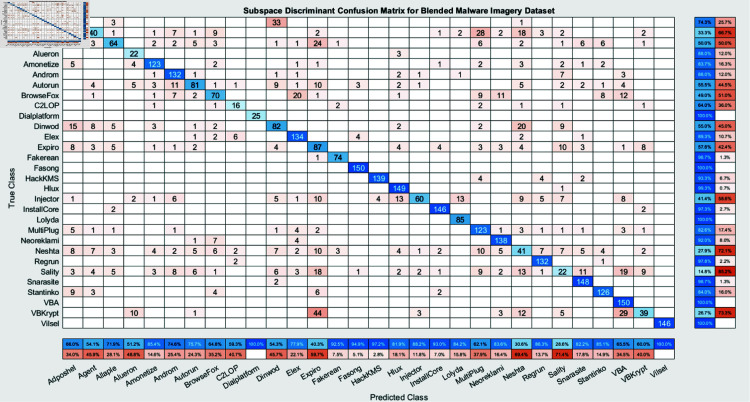
Confusion Matrix for Subspace algorithm (3000 Learning Cycles) providing insight into the performance evaluation of Subspace algorithm.

**Fig 7:**
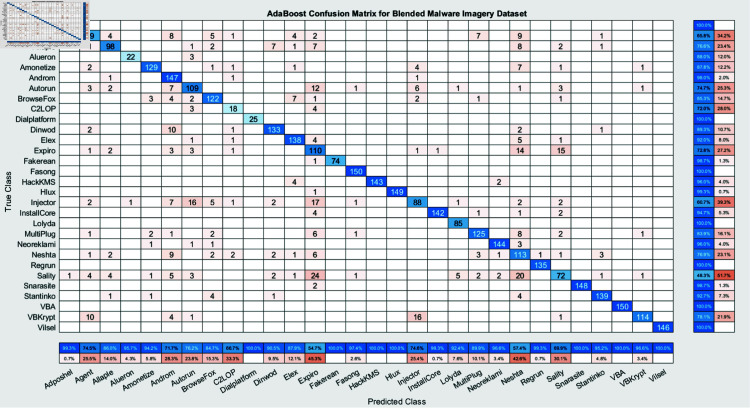
Confusion Matrix for AdaBoost (3000 Iterations) gives detailing regarding the performance in terms of correctly and incorrectly classified instances for each class.

**Fig 8:**
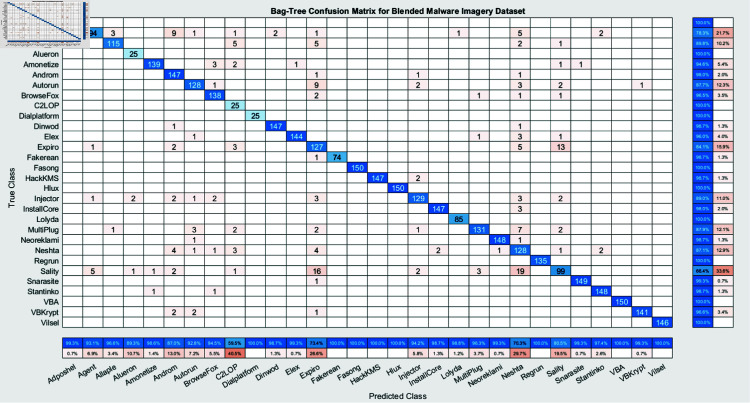
Confusion Matrix for Bagging (3000 Trees) indicates the classification results for the imagery dataset, emphasizing its capacity to correctly classify each class and the degree of misclassifications.

The experimental findings of an ensemble deep neural network with a learning rate of 0.003 across 500 epochs are shown in Figs 2 and 3. It indicates that the network effectively captures the core attributes from the data, which is determined by its excellent accuracy and well-balanced performance metrics. Furthermore, explaining the variations in classification efficacy among these algorithms are the confusion matrices for the different ensemble models: RUSBoost (Fig 4), Random Forest (Fig 5), Subspace (Fig 6), AdaBoostM2 (Fig 7), and Bagging (Fig 8). RUSBoost demonstrates good classification efficiency, effectively regulating class imbalance and eliminating misclassification errors, using 3000 learning cycles (Fig 4).

The term reliability for detecting a range of trends in the information is illustrated by the Random Forest algorithm (Fig 5), which leverages 3000 trees and demonstrates excellent accuracy.

Nevertheless, the Subspace model (Fig 6) exhibits restrictions in this specific use case, with an elevated number of false positives and false negatives and relatively lower performance. After performing over 3000 iterations of training, AdaBoostM2 (Fig 7) attains an appropriate proportion between efficiency and accuracy, beating Subspace but underperforming relative to RUSBoost or Random Forest. Bagging, with 3000 trees (Fig 8), demonstrates constant performance across classes and performs similarly to Random Forest, emphasizing the strength and trustworthiness of ensemble algorithms that accumulate numerous weak learners. Generally, these graphs show the positive traits as well as the limitations associated with each algorithm.

## Explainability analysis and statistical intuitions

The SHAP, LIME, and Grad-CAM visualizations perform a comprehensive explainability analysis, showing statistical intuitions into feature importance and how they impact the prediction of the framework. The SHAP bar graphs of conditional and interventional algorithms demonstrate context-dependent and independent participation of individual features by presenting Mean Absolute SHAP values across different malware categories, consequently evaluating feature importance. This is augmented by LIME explainability, which stipulates instance-specific understandings that underline the local arrangement of feature impact by generating feature, score and feature importance maps. Grad-CAM illustrations provide spatial transparency in the decision-making process by emphasizing and focusing on image areas that are most significant to the prediction of the framework. These approaches collectively present a powerful statistical analytic framework under the explainability umbrella, granting an in-depth understanding of feature contributions and encouraging transparent and explainable malware classification.

### SHAP (SHapley Additive exPlanations) analysis

A common approach for interpreting DL/ML model predictions is providing statistical significance to distinct features, referred to as SHAP (SHapley Additive exPlanations) values. SHAP outcomes, obtained from cooperative game theory, contribute to evaluating the relevance of each feature to a forecast, facilitating the analysis of intricate models. It is especially important to grasp which features impact model decisions across samples or categories for critical applications like malware detection. This is where SHAP explanations can be beneficial.

**Fig 9:**
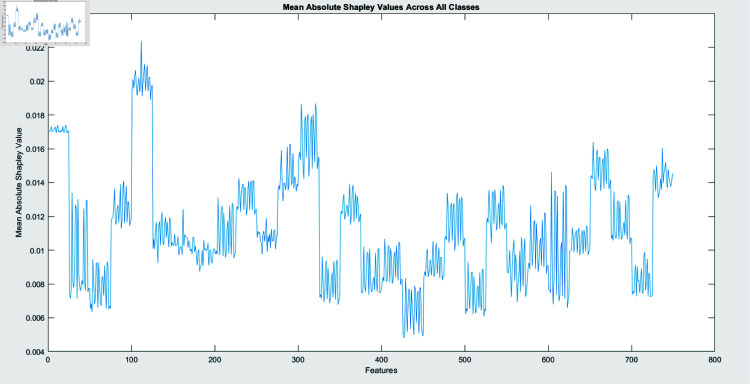
Visualization of mean absolute SHAP values for conditional algorithm across all classes.

SHAP analysis is performed through two algorithms: Interventional and Conditional. The figures, created by an interventional approach (Fig 11) and a conditional technique (Fig 9), evaluate the Mean Absolute SHAP (SHapley Additive exPlanations) values across various malware types. These two graphs represent a global view of feature significance for each malware category across different scenarios by integrating data over several query points (25). Contrary to concentrating on a single instance, this method emphasizes the global behavior of the model once exposed to a large dataset.

**Fig 10:**
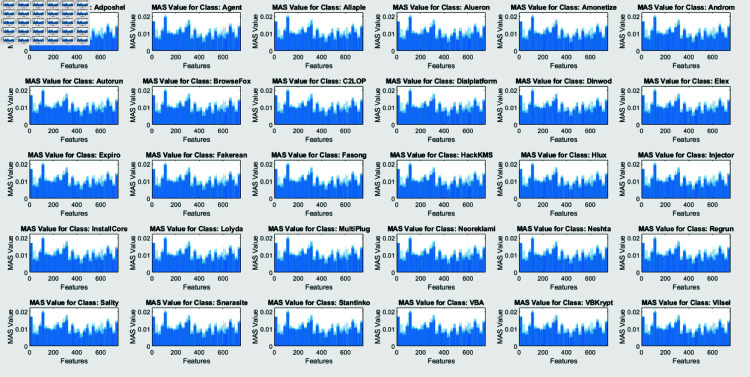
Visualization of mean absolute SHAP values for conditional algorithm for each class.

**Fig 11:**
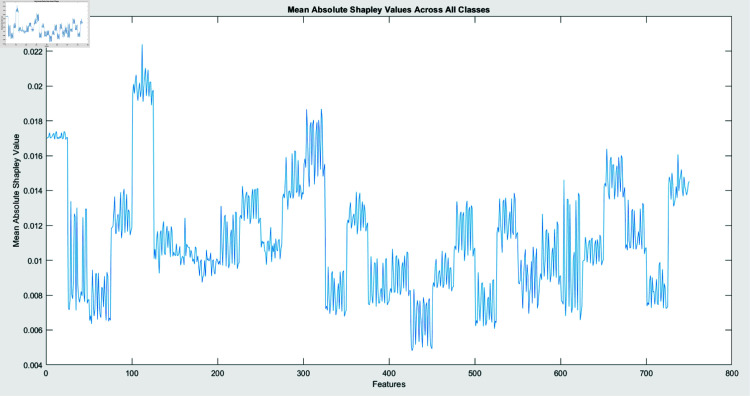
Visualization of mean absolute SHAP values for Interventional algorithm across all classes.

**Fig 12:**
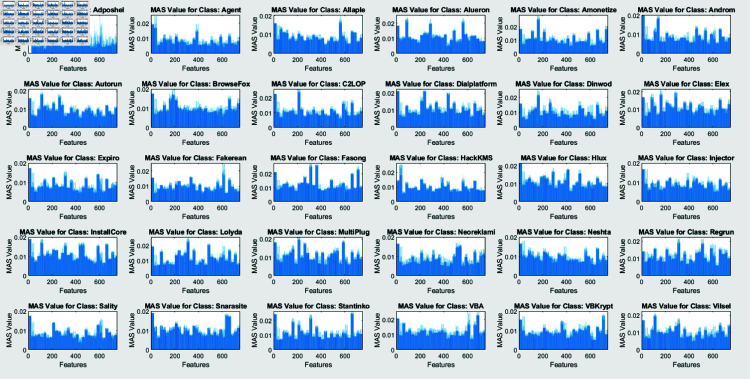
Visualization of mean absolute SHAP values for Interventional algorithm for each class.

The comprehensibility techniques are contrasted in Figs 10 and 12. The conditional SHAP values in Fig 10 satisfy certain conditional dependencies across the dataset while highlighting feature significance. This approach reveals subtle dependencies that may define malware patterns of action by exposing instances in which specific traits assist in identifying malware categories under standard interdisciplinary relationships. Fig 12, revealing interventional SHAP values, presents a distinct approach by examining the effect of each feature independently from these associations. The interventional technique presents a more straightforward, unconditioned evaluation of feature significance with a lower impact through feature relationships by separating the effect of every attribute from the categorized outcome.

These features are significant markers for particular malware classes, irrespective of contextual associations, as evidenced by the inline feature relevance across conditional and interventional SHAP values in Figs 10 and 12. However, fluctuations in feature scores across the two figures reveal that the dependability of the framework on specific elements might vary based on whether conditional dependencies are taken into consideration. This demonstrates the significance of both techniques: Interventional SHAP delivers knowledge of explicit feature contributions while ignoring the effects of contextual dependencies. The conditional SHAP is especially beneficial for interpreting complicated and dependent feature associations.

### LIME explainability

A model-agnostic method called LIME (Local Interpretable Model-agnostic Explanations) estimates the model spatially by employing an explainable model, like linear regression, to interpret distinct predictions. The activity entails generating disruptions to the input data, yielding predictions for the modified data, and subsequently utilizing a fundamental framework to fit the data and resolve which features substantially determine the prediction. Data disturbance, forecasting, applying an explainable model, and feature significance grading are all steps in the LIME process. It recommends thorough knowledge at the feature level, highlighting specific components that impact the prediction of the framework.

**Fig 13:**
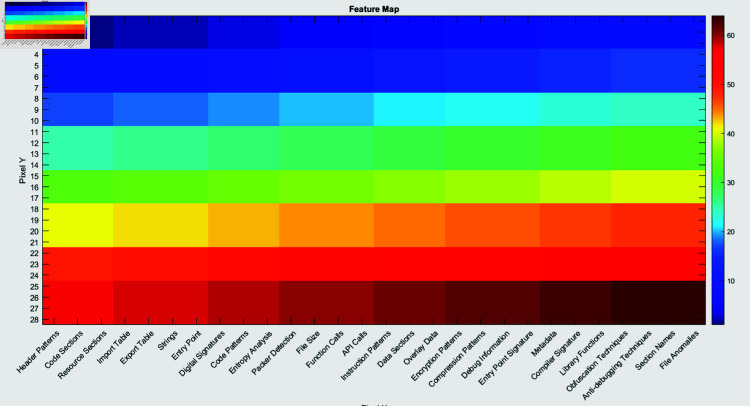
Visualization of the feature map indicates the stimulation of various filters in the convolutional layers, implying how the network observes different features of the input image.

**Fig 14:**
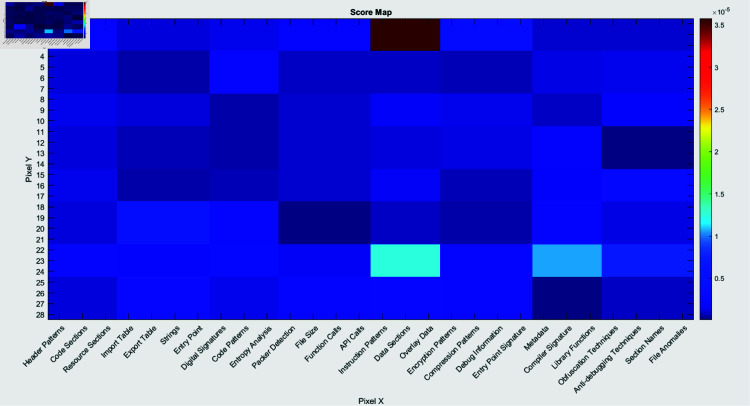
Visualization of the Score Map depicts the regions that contribute the most to the classification score.

**Fig 15:**
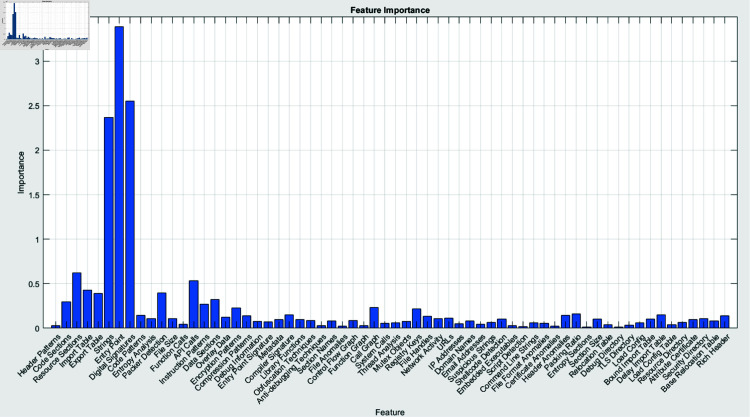
Feature importance interprets the rank of features based on their contribution to the final decision.

Figs 13, 14 and 15 demonstrate the feature map, score map, and feature importance map, respectively, as produced by the LIME approach. The categorized imagery features that facilitate the framework to formulate predictions are projected in the feature map (Fig 13). The areas that participate the most in the classification score are emphasized in the score map (Fig 14).

The features are categorized in the feature importance map (Fig 15) according to their significance in the prediction process. These illustrations deliver an extensive comprehension of segments and features of the image that contribute to the model for classification, which are depicted in Table 3. These prominent features demonstrated important details regarding the way malware may endeavor to avoid tracking, maintain perseverance, or interface with other platforms.

**Table 3:** Description of features.

Feature	Description
Header Patterns	Comprises of those features that may determine the type and behaviour of the file.
Code Sections	Significance in determining the area where functionality resides.
Resource Sections	Resources like visuals, characters, or additional details, that may possess harmful information embedded in them
Import Table	List of modules or packages that have been imported, demonstrating prevalent behaviour and related dependencies.
Export Table	List of functions or symbols made available to other programs, potentially revealing intended interactions.
Strings	Human-readable text in the file, such as file destinations, web addresses, or malware-related terms.
Entry Point	The position from where the executable begins to run; inspection of this part can expose strategies for deception or exploitation.
Digital Signatures	Signatures based on credentials guarantee the record's validity or source; lacking them may lead to distrust.
Code Patterns	These trends may emphasize specific malware types or behaviors.
Entropy Analysis	High entropy can represent encoded or compressed material to gauge a degree of randomness in file sections.
Packer Detection	Identifying encrypted or restrictive techniques known to mask code prevalent in malware.
File Size	The executables regardless of the size may represent an indicator of limited payloads or dubious compression.
Function Calls	Behaviours like file tampering, communication, or durability can be accessible by system or API calls.
API Calls	The software executes certain system API functions that help detect illicit activities.
Instruction Patterns	This computational code or assembly structure and execution designs are indicators of vulnerabilities or illicit behaviour.
Data Sections	Data required by the application is typically located in sections that consist of integrated files or programmed specifications.
Overlay Data	It is common practice to mask payloads or setup files by inserting supplementary data into the final section of the executable.
Encryption Patterns	To keep secrets or preserve fraudulent code by employing sequences that show encryption operations.
Compression Patterns	Compression-indicating sequences can be employed for disguise and encoding.
Debug Information	Expose diagnostic indicators or data that might cover up the activity of the executable or expose how it was written.
Entry Point Signature	Discovering recognised viruses or benign programs becomes easier by unique characteristics at the starting point.
Metadata	Timestamps and author details are instances of such file data that may demonstrate alterations or interference.

### Grad-CAM explainability

**Fig 16:**
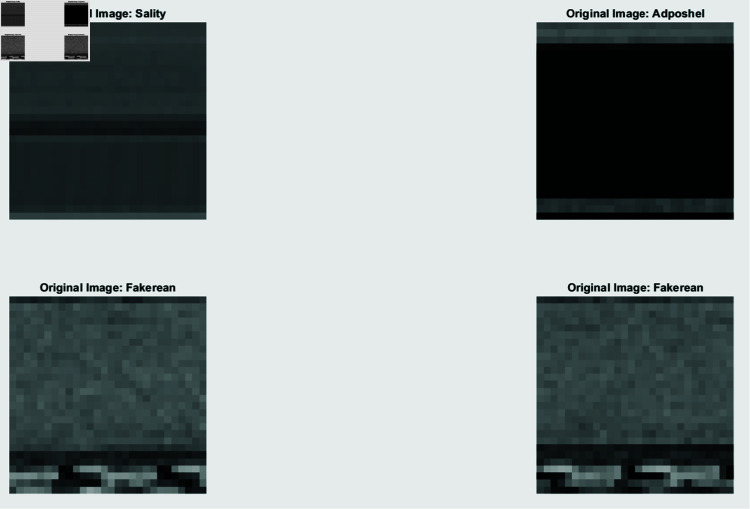
Score map augmented with Grad-CAM for a specific sample image.

**Fig 17:**
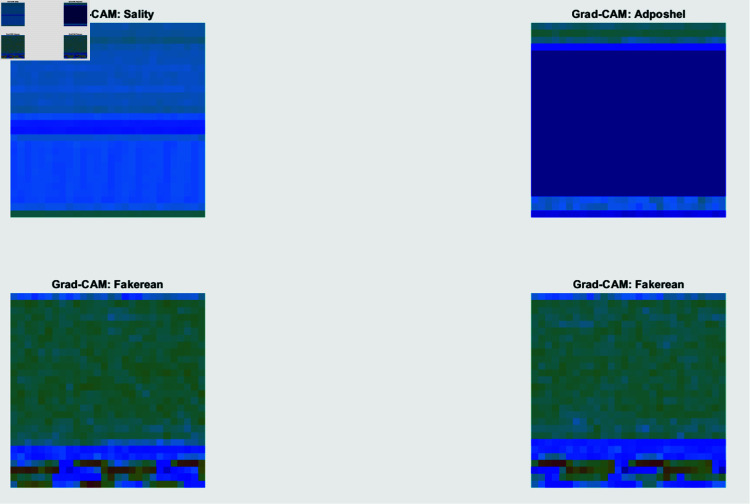
Grad-CAM visualization focuses on the areas of the image that impacted the prediction of the model the most.

The Gradient-weighted Class Activation Mapping (Grad-CAM) technique implies calculating the gradient of the class score concerning the feature maps of a convolutional layer. This practice enables the revelation of certain regions within an input image that performs a key function in the classification decision. A heat map emphasizing significant areas of the illustration is the result. The technique involves the choice of a convolutional layer, estimating gradients, implementing global average pooling to attain significant weights, subsequently weighting the feature maps, and utilizing the Rectified Linear Unit (ReLU) to produce the heatmap. Grad-CAM assists in identifying the areas of attention for the model, offering feature awareness, such as headers or code patterns, which are essential for the classification. This improves confidence in the model by confirming adjustment with domain knowledge. Fig 17 exhibits the Grad-CAM justifications of the four sample original images from the Blended Malware Dataset, depicted in Fig 16. The Grad-CAM illustrations superimpose heatmaps onto the original images, demonstrating the focus areas for the model for formulating its classification decision. This aids in illustratively authenticating that the concerned point of the model aligns with familiar malware behaviors and formations.

Grad-CAM and LIME evaluate both interpretability techniques and stipulate complementary verdicts. Grad-CAM emphasizes significant regions in the input image to offer a high-level, visual justification that is beneficial for visual examination and interpreting the graphical focus of the model. LIME, on the other hand, delivers a comprehensive, feature-level explanation by identifying specific elements and their importance for the prediction. This exhaustive exploration assists in understanding the influence of local features on the prediction process of the model. By integrating these approaches, DL models for malware detection become more explainable and trustworthy, enhancing the efficacy and understandability of cybersecurity solutions.

## Conclusion and future work

This study explores ensemble models, including an exclusive deep neural network, in the context of the explainability and efficacy of malware imagery datasets. The deep neural network established its feature extraction and classification dominance by obtaining outstanding accuracy. It is trained with a learning rate of 0.003 across 500 epochs. RUSBoost and Bagging, two of the ensemble techniques, are revealed to be relatively effective at managing class imbalance while offering reliable findings for classification. On the other hand, the efficiency of the Subspace algorithm is lower, demonstrating that it may not be regarded as adequate for this kind of data. By balancing the better versus middling, less effective models, the results of AdaBoostM2 are mediocre.

Explainability assessments are directed by employing illustrations such as feature map, score map and feature importance map of LIME, and Grad-CAM graphical representations. The score map offered details regarding the confidence levels of the model across different categories, while the feature map highlighted the ability of the neural network to separate relevant features. Grad-CAM representations highlighted the regions in the sample images that contributed the most to the model predictions, and the feature importance representation scored the significance of different characteristics in the model’s decision-making process. The SHAP analysis is performed through interventional and conditional algorithms to provide a global perspective on the behavior of the model highlighting which features consistently impact across various samples.

Integrating performance metrics and explainability techniques underscores the importance of reliability and comprehensibility in evaluating mode. The findings indicate that while RUSBoost and Bagging are fairly effective models for this dataset, the deep neural network performs exceptionally outstanding in terms of accuracy and also offers a clear understanding of its decision-making process through graphical explainability. Its excellent efficiency and transparency combine to make it an effective malware detection tool. Future exploration may converge on improving these algorithms and investigating alternative approaches for augmenting the accuracy and explainability of the models employed in the malware classification projects.

## Computing and data resources

The experimental results were generated by utilizing the 7th-generation Core i5 machine supplied with 16 GB of RAM. The evaluation is performed using Matlab 2022a with its Deep Learning Toolbox within a Windows 11 environment. Dataset URL: https://www.kaggle.com/datasets/gauravpendharkar/blended-malware-image-dataset
